# Sheet‐Size‐Dependent Mosaicity of 2D Phyllosilicate Membranes

**DOI:** 10.1002/advs.76913

**Published:** 2026-07-31

**Authors:** Min A Kim, Paul A. Fenter, Sang Soo Lee, Katrina I. Sparks, Yining Liu, Jeffrey W. Elam, Seth B. Darling

**Affiliations:** ^1^ Advanced Materials for Energy‐Water Systems Energy Frontier Research Center Argonne National Laboratory Lemont Illinois USA; ^2^ Chemical Sciences and Engineering Division Argonne National Laboratory Lemont Illinois USA; ^3^ Applied Materials Division Argonne National Laboratory Lemont Illinois USA; ^4^ Pritzker School of Molecular Engineering University of Chicago Chicago Illinois USA

**Keywords:** laminar membranes, phyllosilicates, sheet size, structure‐property relationship, two‐dimensional materials

## Abstract

Two‐dimensional (2D) materials are physical building blocks of laminar membranes with interlayer channels for ion and molecular transport. Here, we systematically investigate the influence of lateral sheet size on the ion permeability and structural organization of vermiculite membranes. We show that the sheet size plays a significant role in governing the microstructure of these laminar systems. High resolution x‐ray diffraction analysis reveals changes in mosaic distribution and vertical domain size depending upon exfoliate lateral size, leading to changes in the degree of polycrystallinity of the membrane. As these structural variations influence ion transport processes through the membrane, we highlight the need of careful size control of exfoliated 2D materials and structural disorder analysis for 2D laminar membrane development.

## Graphical abstract

This study explores how the size of 2D vermiculite sheets affects the structure of laminar membranes. High‐resolution x‐ray diffraction reveals how sheet size governs structural disorder and crystallinity. These changes directly impact ion permeability. The results emphasize the importance of polycrystalline structural analysis and sheet size control when designing 2D laminar membranes.

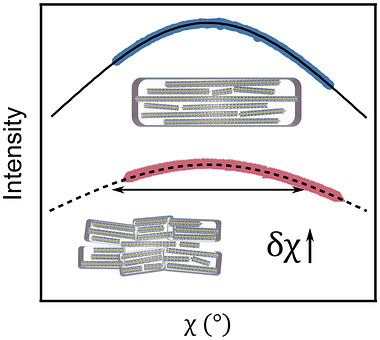

## 1 Introduction

Two‐dimensional (2D) material membranes have emerged as an important class of separation materials [1, 2, 3, 4]. The 2D membranes leverage unique properties of 2D materials such as (near‐) atomic thickness, robustness, functionalizability, and impermeable surfaces, to precisely engineer nano and sub‐nanometer channels, which is a challenging task for conventional membranes [5, 6]. The 2D membranes are generally classified as either nanoporous atomically thin membranes or laminar membranes. While atomically thin membranes offer extremely high permeability and selectivity, their practical implementation is often limited due to scalability challenges [7, 8]. The 2D laminar membranes overcome these limitations by utilizing the self‐assembled interlayer spaces between stacked nanosheets to create highly scalable and uniform transport channels [2, 9, 10]. Owing to their easily accessible fabrication methods along with stable transport pathways and molecular level tunability, various 2D materials have been fabricated into laminar membranes, such as graphene oxides (GO) [11, 12], MXenes [13, 14], layered double hydroxides [15], and phyllosilicates [16, 17, 18, 19]. Naturally occurring phyllosilicate minerals are particularly of interest as attractive alternatives to synthetic 2D materials. Many phyllosilicate minerals can be exfoliated into high‐aspect‐ratio nanosheets due to their intrinsic layered structures, while offering inherent sustainability, low cost, and considerable structural and chemical diversity [20, 21, 22]. Their advantages provide a testbed for scalable membrane fabrication without relying on resource‐intensive materials.

Over the past few years, there has been an increasing effort toward engineering phyllosilicate minerals into functional membranes [19]. For example, various strategies have been studied to lock in interlayer structure to stabilize membranes in aqueous environments, since interlayer spacing defines accessible nanochannel dimensions. Interlayer binding can be enhanced through electrostatic interactions with cations and small molecules [23, 24, 25], and covalent bonding using inorganic pillars [26]. More recently, high‐valence intercalants such as zirconium(IV) and lanthanum(III) ions were shown to become effectively unexchangeable, giving tunable monovalent ion selectivity [27], and deep dehydration of the interlayer has been used to suppress reswelling and sharpen Li^+^/Mg^2+^ selectivity [28]. Furthermore, the interlayer space can be tuned by introducing various species to change the accessible free space within the interlayer channel or the surface charge of the channel walls. Charged copolymers introduced into montmorillonite channels reversed the usual Na^+^/Mg^2+^ migration order and raised mono/divalent selectivity [29]. Intercalated titanium dioxide nanoparticles widened the vermiculite interlayer from 1.35 to 1.42 nm and increased water flux while retaining dye rejection [30], and cobalt nanoparticles expanded it further, from 1.32 to 1.49 nm, opening additional transport paths for higher permeance [31]. In contrast, even for the broader family of 2D materials, the lateral size of exfoliated 2D sheets has received comparatively less attention, although it is one of the important structural descriptors of composed membranes. The geometry of individual sheets can impact the interlayer alignment, which is correlated with the continuity of transport channel pathways, yet its influence on membrane structure and ion permeation remains fragmented and poorly understood. In particular, the stacking structure of laminar membranes has not been examined systematically as a function of lateral sheet size, and the diffraction signatures that do vary with size have largely been read from a single peak that narrows for larger sheets, without separating the peak width into contributions such as the coherent stack height and the in‐plane domain size, or resolving the mosaic distribution of the domains. Those studies showed larger sheets can promote extended structural ordering to achieve precise molecular sieving and better retention performance [32, 33], yet ion selectivity has been reported to be independent of sheet size in some studies [34, 35, 36]. Smaller sheets have been used to leverage shorter and less tortuous transport pathways to improve the membrane permeance without sacrificing their selectivity and rejection rate [13, 36]. Varied d‐spacing and chemistry of edge and basal plane between different materials further add to the complexity of deciphering sheet size effects.

These sheet‐size‐dependent structural variations directly affect transport pathways and, consequently, membrane performance; a better understanding of the microstructure of laminar membranes is essential for establishing generalizable strategies that extend across 2D laminar membrane systems. In this study, we fabricated vermiculite laminar membranes using different, controlled lateral sizes of exfoliated sheets and evaluated their ion permeation behavior and structural characteristics. By analyzing structural information obtained from high‐resolution x‐ray diffraction (XRD), we revealed how sheet size affects layer packing, leading to structural disorder in membrane crystallites that correlate with ion permeability. We show that lateral sheet size changes the mosaic distribution of the stacked layers in membranes as well as their domain sizes, emphasizing the importance of a previously underappreciated design parameter for 2D laminar membranes.

## 2 Results and Discussion

### 2.1 Controlled Size of Exfoliated Vermiculite 2D Sheets

To fabricate vermiculite laminar membranes (VMs), raw vermiculite was exfoliated using a cation exchange swelling technique. The cation exchange process weakens the interlayer interactions within the vermiculite structure by ion intercalation, reducing the force required to exfoliate the individual layers. Following the cation exchange, the expanded vermiculite was subjected to gentle sonication, which served a dual purpose: acting as a physical exfoliation force to separate the layers and as a breakdown method to further control the lateral size of the exfoliated vermiculite sheets.

**FIGURE 1:**
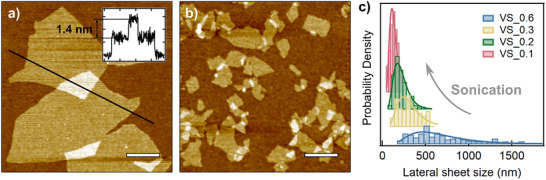
Exfoliated single‐layer vermiculite sheets. AFM image of exfoliated vermiculite after (a) 1 h and (b) 25 h of ultrasonication. Scale bar is 400 nm, and the *z*‐axis contrast is adjusted to show the same scale of ± 3.15 nm. Inset in (a) shows cross‐sectional height trace corresponding to the black solid line in (a). (c) Probability density distribution of lateral length of exfoliated vermiculite sheets with different sonication degree (1, 4, 10, and 25 h represented in blue, yellow, green, and red, respectively). The median sheet sizes were 0.6, 0.3, 0.2, and 0.1 µm from 1 to 25 h samples.

We first prepared an exfoliated vermiculite solution by sonicating a cation‐exchanged vermiculite solution for 1 h, followed by centrifugation to separate unexfoliated multilayer vermiculites. Figure 1a shows an atomic force microscopy (AFM) image of exfoliated vermiculite sheets from this initial vermiculite solution (VS_0.6). The cross‐sectional height corresponding to the black line in Figure 1a showed the 1.4 nm step height between the stack of two individual layers of vermiculite. This height agrees well with a single layer of vermiculite consisting of a talc‐like layer (9.26 Å) with a water layer (4.97 Å) [37], demonstrating that the exfoliated sheets were predominantly single layers after the exfoliation process. The lateral size of exfoliated vermiculite was approximated by the means of the major and minor axes of individual flakes. The size survey showed a broad range of vermiculite sheet size from 0.2 to 2 µm with a positively skewed distribution.

A series of vermiculite solutions was prepared with different durations of ultrasonication to further reduce the lateral dimension of exfoliated vermiculite sheets. A representative AFM image of vermiculite sheets after 25 h of sonication is shown in Figure 1b. As previously observed with other 2D materials [38], exfoliated vermiculite sheets are broken into smaller sizes with extended sonication, shifting the size distribution of the exfoliated solution. Vermiculite solution with sonication times of 1, 4, 10, and 25 h (noted as VS_0.6, VS_0.3, VS_0.2, and VS_0.1, respectively) showed a gradual down‐shift of their lateral size distribution profile (Figure 1c). The median length of the vermiculite sheet was reduced from 620 to 130 nm after 25 h of sonication.

### 2.2 Fabrication of Laminar Membranes With Different Vermiculite Sheet Size

**FIGURE 2:**
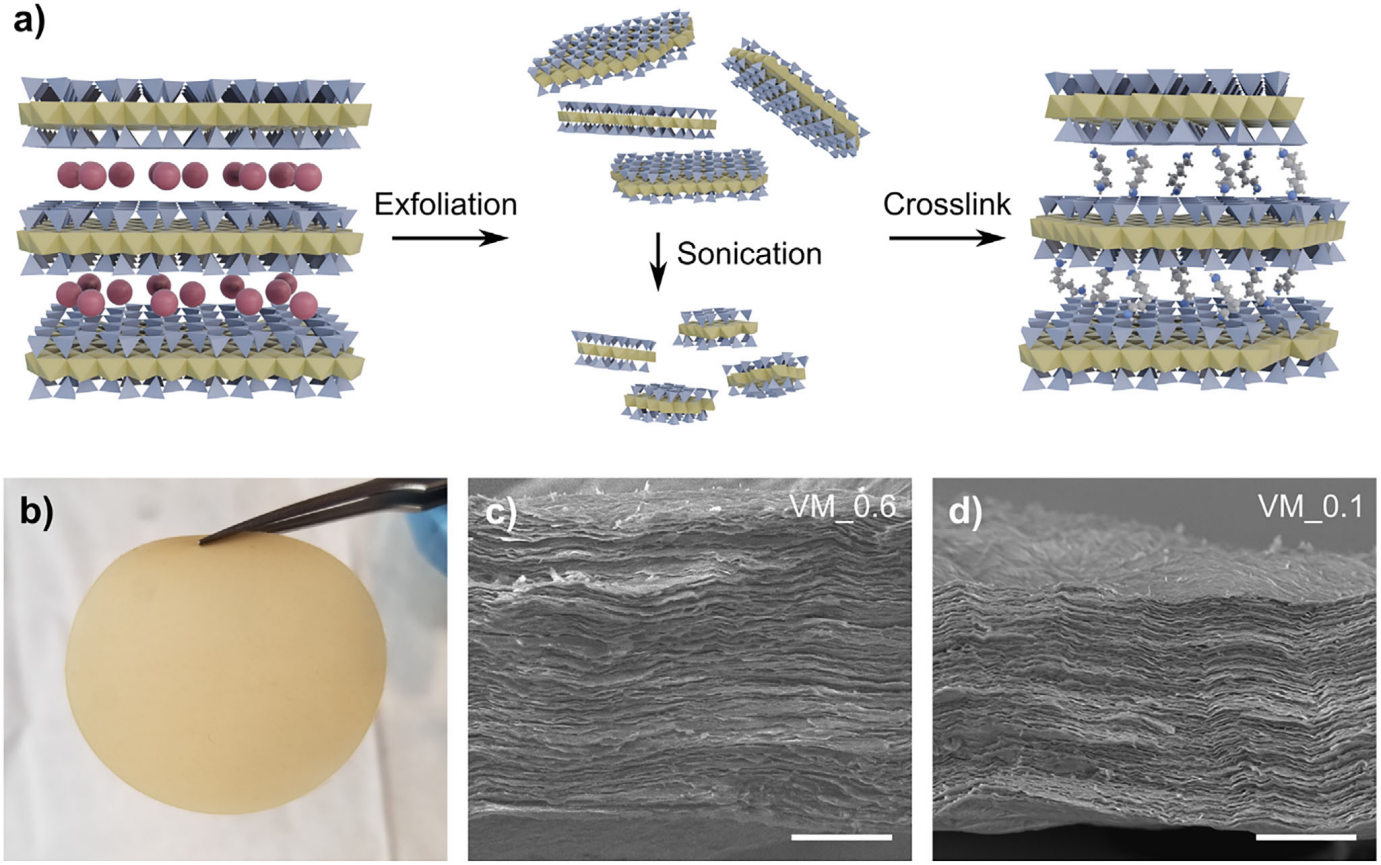
Fabrication of 2D laminar membranes with vermiculite. (a) Schematic representation of membrane fabrication steps. (b) Photo of typical vermiculite membrane. SEM images of a membrane cross‐section fabricated with (c) large vermiculite sheets (∼0.6 µm) and (d) small vermiculite sheets (∼0.1 µm). Scale bar is 10 µm.

Once exfoliated vermiculite at different sheet sizes were prepared, those exfoliated sheets were re‐stacked into laminar membranes using vacuum filtration. A schematic of the vermiculite membrane fabrication steps is shown in Figure 2a. For all membranes in this study, a diamine solution (1,6‐hexanediamine) was used as an organic linker, which has been previously reported to control the interlayer distance while enhancing the water stability of vermiculite membranes [23]. A picture of a typical vermiculite membrane fabricated with this process is shown in Figure 2b. The free‐standing VMs are not only mechanically stable in water, but their mechanical flexibility also provides easy handling. Obtained VMs showed consistent water stability and mechanical robustness regardless of the size of the vermiculite sheet used.

The membrane's macrostructural arrangement is often confirmed with cross‐sectional scanning electron microscopy (SEM). Figure 2c,d show SEM images of cross‐sections of VMs (VM_0.6 and VM_0.1) fabricated with large (VS_0.6, $\tilde{x}$ = 0.6 µm) and small (VS_0.1, $\tilde{x}$ = 0.1 µm) exfoliated sheets, respectively. Both membranes showed a distinct layered structure, typical evidence of a well‐ordered 2D laminar membrane where the individual sheets are expected to be oriented parallel to each other from layer‐by‐layer stacking action to form the macrostructure shown. The effects of the exfoliate lateral size were not evident in the cross‐sectional images.

### 2.3 Effect of Sheet Size on Ion Permeation

**FIGURE 3:**
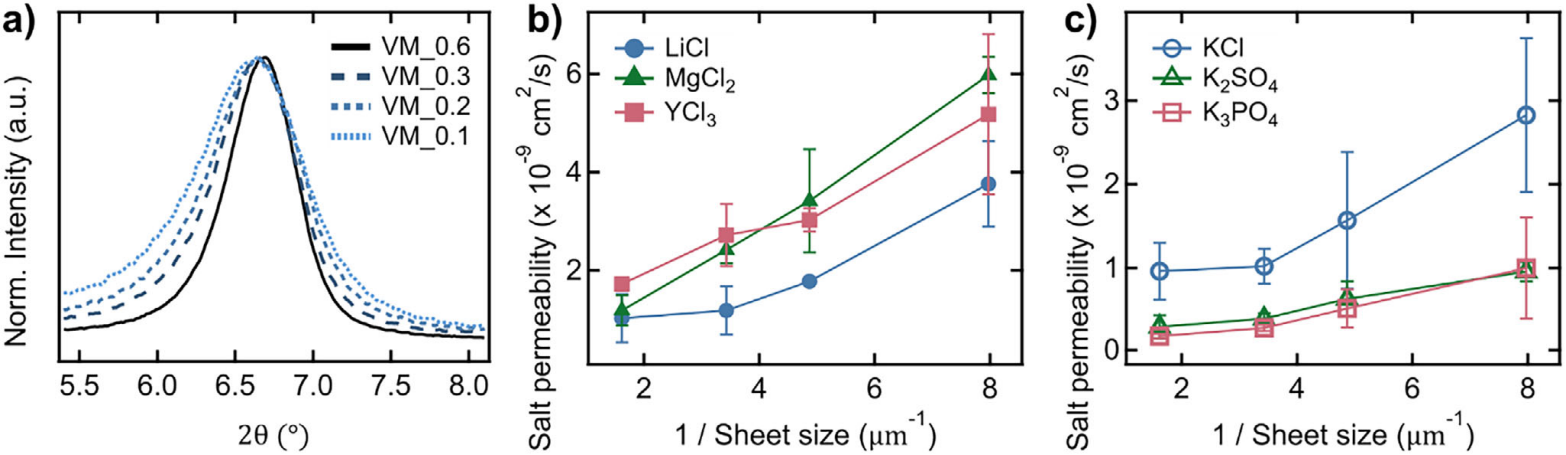
Permeability of salt solutions through vermiculite membranes made with different sheet sizes. A series of vermiculite membranes (VM_0.6, VM_0.3, VM_0.2, and VM_0.1) was fabricated using vermiculite solutions with the different median sheet sizes (0.6, 0.3, 0.2, and 0.1 µm, respectively). (a) XRD 2θ scan of vermiculite membranes measured with a laboratory XRD system. Permeability of (b) cations and (c) anions through vermiculite membranes as a function of the inverse of the sheet size.

Restacked vermiculite exfoliates showed similar interlayer spacing as measured by the vermiculite (001) Bragg peak position as a function of scattering angle, 2θ. A series of VMs were prepared with different exfoliate solutions from Figure 1c. As shown in Figure 3a, the position of the Bragg peak varied only slightly from VM_0.6 to VM_0.1, indicating that the interlayer spacing of fabricated membranes remained similar while the median lateral length of vermiculite sheets decreased by ∼80%. Water‐soaked membranes measured after 24 h soaking also showed a similar variation (Figure S1). The d‐spacing variation across the membrane series was 0.1 to 0.3 Å, consistent among repeated measurements. Slight shifts of the Bragg peak position may arise from minor differences in residual interlayer species between membranes.

On the other hand, a systematic broadening of the (001) reflection was observed across the membrane series, suggesting a decrease in the effective crystallite size along the stacking direction. As the full width at half maximum (FWHM) of a Bragg peak is inversely proportional to the size of the crystalline domains, the increase of FWHM from 0.52° to 0.81° indicates a decrease in the number of coherently diffracting vermiculite layers within the individual stacks, corresponding to a reduction in the effective crystalline domain sizes from ∼17 to ∼11 nm (i.e., ignoring the role of instrument resolution and non‐ideal crystalline order), a ca. 36% decrease across the membrane series. This peak broadening suggests that the decrease in the lateral size of the vermiculite sheets also led to a reduction in the quality of vertical stacking of vermiculite sheets, indicating a higher degree of structural disorder within the membrane.

One might expect the ion permeation behavior to be similar if the interlayer distance is comparable, providing similar vacant space in the channel. It is a reasonable assumption that their chemical properties remain the same, as there is not a significant change in defect density on the surface of vermiculite layers within 1–25 h of sonication time. While prolonged sonication can introduce defects in certain 2D material sheets [39], these are unlikely to add transport pores in the case of vermiculite. Each layer is a 2:1 structure with an octahedral sheet between two tetrahedral silicate sheets, so a basal‐plane vacancy is unlikely to perforate its ∼1 nm thickness, unlike in single‐atom‐thick materials. The membrane composition was also characterized with x‐ray photoelectron spectroscopy (Figure S5). The silicate signal and the linker‐derived nitrogen were comparable across the four membranes, with no systematic change across the membrane series. The Si/O ratio was also comparable across the series at all etch depths, consistent with no measurable increase in framework vacancies with sonication time. The N 1s region was fit by the same two components, near 399.8 and 401.6 eV, assigned to neutral and protonated amine, respectively (Figure S6). Peak positions were stable to within 0.3 eV, and the component area ratio was near unity across the membranes, suggesting that the linker‐driven chemical environment in the interlayer is largely similar across the series.

To explore the effect of sheet size on the ion transport behavior of a laminar membrane, we performed a diffusion dialysis experiment in an H‐cell using the concentration gradient as the driving force to measure ion permeability. Various 0.1 m salt solutions (LiCl, MgCl_2_, YCl_3_, KCl, K_2_SO_4_, and K_3_PO_4_) were used as feed solutions, and the permeate side was filled with the same volume of DI water. Permeate aliquots were collected periodically to monitor the ion concentration using inductively coupled plasma—optical emission spectrometry (ICP‐OES). The permeabilities for different ions were obtained under steady‐state diffusion conditions using Fick's first law.

The ion permeability for cations with various valences and sizes exhibits several trends. Species transported through interlayer channels experience two separation mechanisms, namely, size exclusion and Donnan effects (electrostatics). When the ion‐transport process is governed by size exclusion, one can expect Mg^2+^ or Y^3+^ with a larger hydrated radius to have relatively lower ion permeability. However, as previously reported [23, 24, 33], higher Mg^2+^ or Y^3+^ permeability was observed compared to Li^+^ and K^+^, as shown in Figure 3b. When cations pass through the nanochannels of VMs, the negatively charged vermiculite interlayer channels have strong electrostatic interactions upon them as the channel scale is on the order of the Debye length (ca. 1 nm). Compared to monovalent cations, Mg^2+^ or Y^3+^ are expected to pass more readily due to stronger attraction to the negatively charged membranes according to the Donnan effect. Therefore, electrostatic interaction is likely to play a larger role overall in cation transport through these vermiculite membranes in comparison to size exclusion. Trivalent cation Y^3+^ exhibited lower permeability than Mg^2+^ when the sheet size of the membrane became smaller, yet it had higher permeability in those with longer sheet size. The reversal in permeabilities between these two cations may reflect an entrance effect linked to the more fragmented domains of the smaller sheet membranes. The broadening of the (001) reflection indicates smaller coherent domains, and therefore more frequent interruptions of the interlayer channels along the transport path. As the sheet size decreases, there are increased opportunities for each ion to enter/exit an interlayer channel while the time it spends inside each channel is reduced. Those gaps between channel exit and entrance could induce the formation of new water layers around Y^3+^ ions before they re‐enter the next interlayer channel, which would require ions to partially or fully shed the second water shell [40]. Because the second hydration sphere of Y^3+^ is slightly larger than the hydration radius of Mg^2+^ [41], this step weighs more heavily on Y^3+^ and raises the role of size exclusion for that ion where channel interruptions are most frequent, consistent with the reversal appearing only in the smaller‐sheet membranes. The steady‐state permeabilities locate this crossover but do not isolate its origin, and a temperature‐dependent measurement of the transport activation energy could be conducted to confirm the mechanism [42].

The ion permeability for anions exhibits a general trend of Cl^−^ > SO_4_^2−^ > PO_4_^3−^ (Figure 3c). The fast diffusion of chloride ions compared to sulfate and phosphate ions suggests that transport is governed in part by a size‐exclusion process, as hydrated chloride ions have the smallest size among this set. On the other hand, sulfate ions have a slightly larger hydrated ionic radius in comparison to phosphate, which suggests that Donnan effects also contribute to the ion transport process. VMs have a repulsive surface charge property for anions. Therefore, the larger negative charge of the multivalent anions (phosphate ion) will experience more repulsive electrostatic interactions [5, 43].

Interestingly, the ion permeability of each individual ion shows a consistent trend of having increased permeability with decreased vermiculite sheet size regardless of the charge polarity. Such observation cannot be explained with the interlayer channel distance in the laminar membrane, since the change in d‐spacing measured by XRD analysis was small relative to the increase in permeability and did not track sheet size, as shown in Figure 3a. The physical distance between stacked layers of vermiculites is still governed by the molecular cross‐linker; therefore, membranes should have similar size exclusion effects dictated by the interlayer channel space. However, the observed broadening of the (001) reflection suggests a change in the membrane morphology toward a more fragmented architecture. This shift follows the observed diffusion trend that all ions show a strong negative correlation between their permeability and the lateral size of vermiculite sheets in the membranes, which suggests additional transport pathways outside of the interlayer space that we need to consider. Beyond permeability, this size‐dependent structural change also affects ion separation. Binary ion system measurements showed that the K^+^/Mg^2+^ selectivity increased with decreasing sheet size (Figure S3). The increase was driven mainly by the rising K^+^ permeability toward smaller sheets, while the Mg^2+^ permeability remained low. This is consistent with a more fragmented architecture at a smaller sheet size, opening additional pathways that the weakly hydrated K^+^ could access more readily than the more strongly hydrated Mg^2+^, if entering those pathways requires partial shedding of the hydration shell. This points to lateral sheet size as a possible structural handle for tuning ion selectivity.

### 2.4 Membrane Structural Analysis With High‐Resolution XRD

**FIGURE 4:**
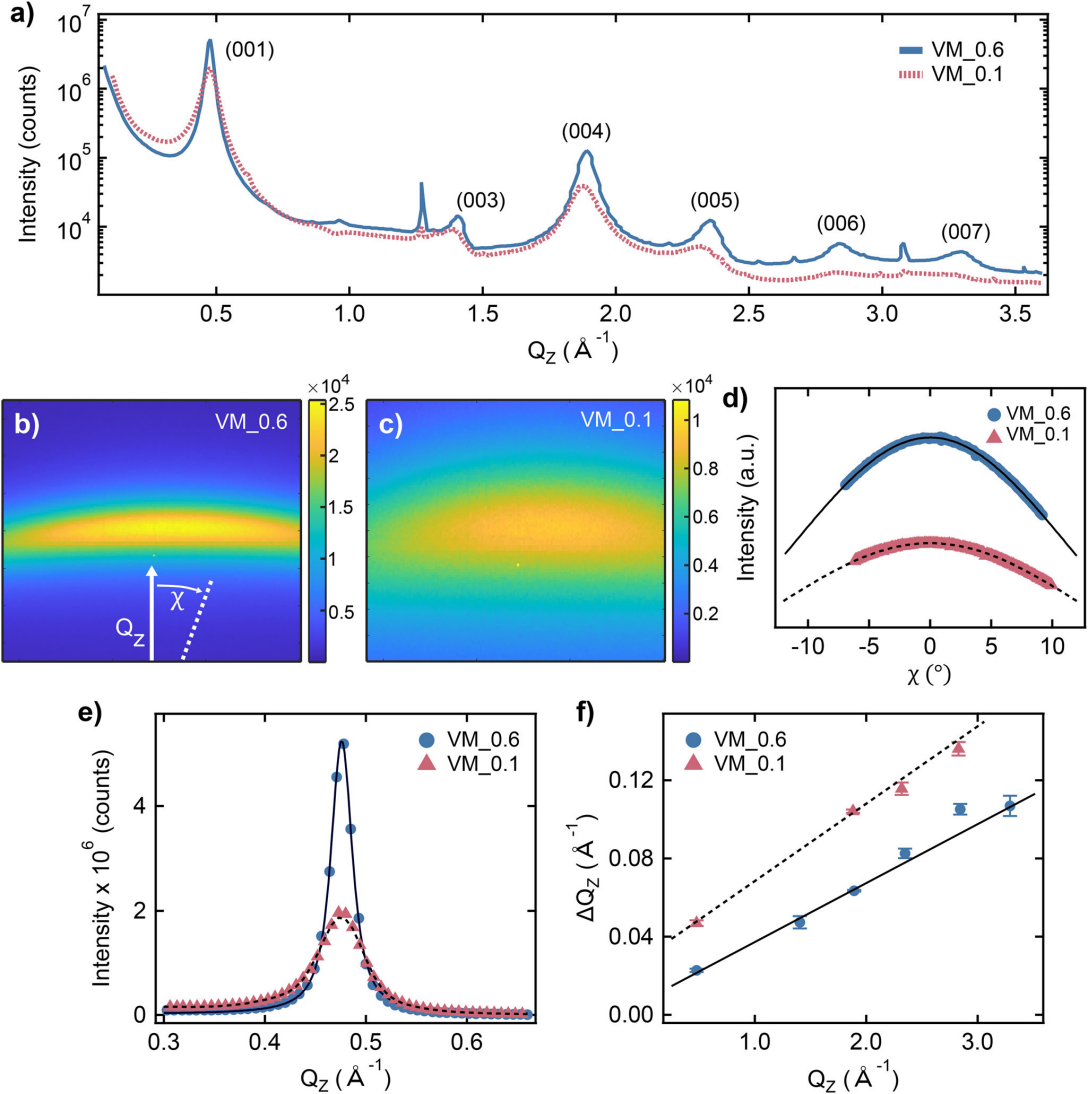
Analysis of structural disorder in vermiculite membranes with high‐resolution XRD. (a) Wide‐angle synchrotron x‐ray diffraction data for VMs with different sheet sizes (VM_0.6 in blue solid line and VM_0.1 in red dashed line). Detector images showing different diffraction peak shapes at the (001) reflection with the momentum transfer, Q_z_, along the vertical direction and the mosaic disorder in the lateral direction, characterized by the angle, χ, for the membranes (b) VM_0.6 and (c) VM_0.1. (d) Mosaic width of the (001) reflection along with fits to the mosaic width. (e) Vertical diffraction line profiles and corresponding line‐shape fits for the (001) reflection as a function of Q_z_. (f) The variation of the diffraction peak width for multiple (00L) reflections consistent with the presence of lattice strain. VM_0.6 is shown in blue circle with solid line fit, and VM_0.1 is shown in red triangle with dashed line fit.

To better understand the structural disorder within the vermiculite membranes, we performed synchrotron‐based x‐ray diffraction at beamline 7‐ID‐C at the Advanced Photon Source. The XRD data (Figure 4a) show multiple (00L) reflections that vary over three orders of magnitude in intensity. Consistent with the lab XRD data (Figure 3a), the membrane with small sheet size (VM_0.1) shows broader peak widths, indicating increased disorder compared to the membrane with larger sheet size (VM_0.6). The differences in lattice disorder for the two membranes are visualized using individual detector images at the (001) diffraction condition in Figure 4b,c. These images show that the vermiculite layers are preferentially ordered along the direction normal to the membrane, but that the degree of ordering is intermediate between a single crystal (which would have a diffraction peak that is laterally sharp) and a uniform powder (which would show powder rings with uniform intensities). This disorder can be quantified as a mosaic width (Figure 4d), where we find the membranes VM_0.6 and VM_0.1 to have a FWHM of Δχ = 26.4° and 30.6°, respectively, indicating ca. 16% increase in mosaic width with the sheet size reduction.

A second source of disorder is seen through the systematic variation of the diffraction peak width for the (00L) reflections. The (001) reflection as a function of vertical momentum transfer, Q_z_, with fits to the peak shape, is shown in Figure 4e. The Bragg peak line shape of the (001) reflection was about twofold broader for the VM_0.1 as compared to the VM_0.6, with nominal domain sizes of 13.4 and 27.7 nm, respectively. These crystalline domains are larger than the values shown in Figure 3a because of the higher angular resolution for these synchrotron‐based data. We also observed that the line widths, ΔQ_z_, increased with Q_z_. Consequently, the peak width of the (001) reflection provides only a lower limit on the crystal domain size of the membrane. A line‐shape analysis across a series of the (00L) reflections shows a systematic and linear increase in peak width (Figure 4f). This is consistent with peak broadening due to a combination of finite particle size and strain (e.g., from variations in lattice spacing between crystalline grains) [44, 45, 46]. The average crystalline domain size can then be recovered from a linear fit of the observed peak widths as a function of momentum transfer, as shown in solid and dashed lines in Figure 4f. The extrapolated peak width at Q_z_ = 0 reveals the intrinsic widths from the vertical intercept (VM_0.6 = 0.007 Å^−1^ and VM_0.1 = 0.029 Å^−1^). Based on those widths, the intrinsic crystalline domain sizes were 88.0 nm for VM_0.6 and 21.7 nm for VM_0.1, a fourfold difference in average crystallite size between the membranes.

**FIGURE 5:**
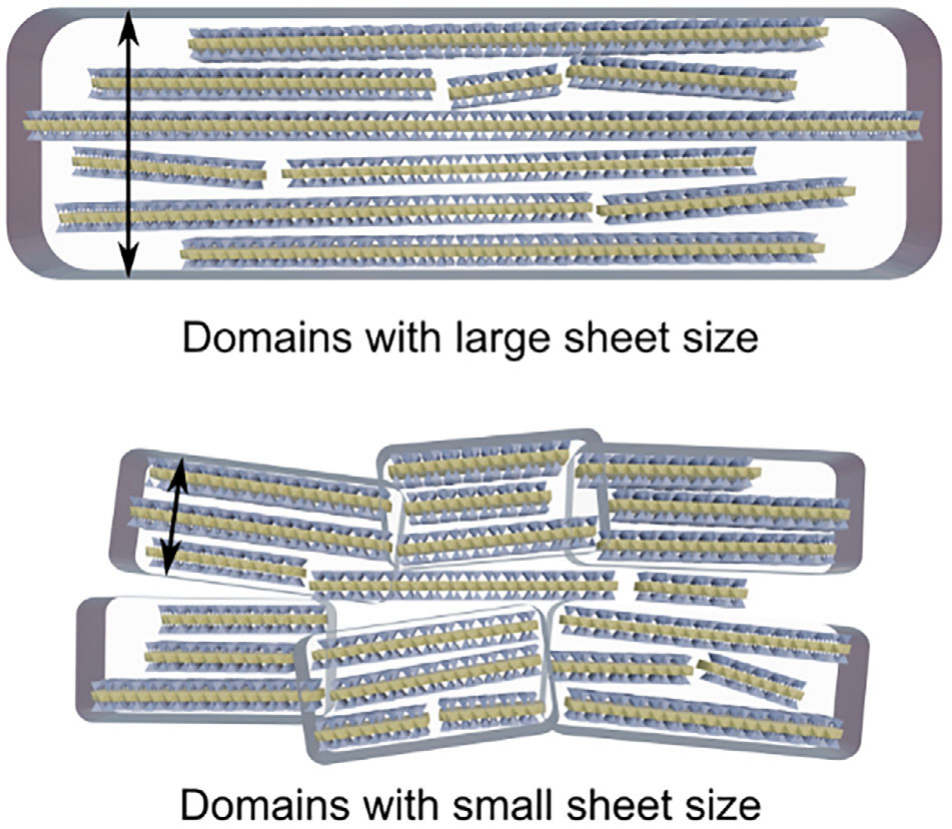
Diagram of microstructure of 2D laminar vermiculite membrane with different sheet size.

The XRD results show that vermiculite membranes can be viewed as polycrystalline materials. The polycrystalline material is composed of misoriented yet relatively defect‐free domains. In vermiculite membranes, the exfoliated individual sheets are stacked to form coherent, ordered crystalline domains within the structure. The stacking distance of vermiculite layers was mainly governed by the cross‐linker used, as a negligible difference in Bragg peak position between membranes confirmed the dominant role of cross‐linker in defining the d‐spacing. From those blocks of re‐stacked exfoliated layers, misoriented domains are formed, which can be represented by a mosaic block model, where the out‐of‐plane rotation of the individual blocks perpendicular to the surface normal is seen as the mosaic width. This disorder reveals the dependence of lateral sheet size, as shown with increased mosaic width with smaller‐sheet membranes. The sheet‐size effect on membrane structure is depicted schematically in Figure 5. During the self‐assembly of membrane layers, there is always a favored laminar stacking of individual sheets, but also, stacking defects happen where a new layer sits at an angle, not perfectly parallel to the 2D plane. The new stack of layers continues to assemble on top of the misfit layer, leading to the formation of distinct domains throughout the membrane similar to polycrystalline‐like mosaic blocks. In the case of a membrane with a relatively large sheet size, there is a lower chance of initial stacking faults with a larger aspect ratio, reducing mosaic tilt distribution.

Notably, the ratio of vertical and lateral dimensions of the crystalline domains (from the XRD line‐shape analysis and AFM results, respectively) was 0.14 and 0.17 for VM_0.6 and VM_0.1, respectively. Therefore, even though the lateral particle dimension was reduced by a factor of 5 during sonication, a similar reduction in vertical crystallite size was also observed, suggesting that the aspect ratio of the individual grains in the vermiculite membrane is largely unchanged by the reduced exfoliate size. The consistent vertical and lateral aspect ratios across the different grain sizes suggest that the intrinsic grain boundary network remains geometrically similar, while the total volume fraction of those boundaries within the structure changes. This change in the grain boundary density can impact mechanical properties and surface energy of the bulk membrane, as well as ion mobility with intergranular diffusion. The grain boundaries can act as separate channels for ion transport in addition to the interlayer channels within the layered domain. When their density is higher, as in the smaller‐sheet membranes, a larger fraction of these boundary channels is available, which would be expected to shorten the effective path an ion travels and reduce the membrane tortuosity. This runs opposite to classical platelet models, where impenetrable layers with denser packing raise tortuosity and lower permeability [47, 48], the difference being that the grain boundaries here act as conductive pathways rather than barriers. This offers a possible structural account for the higher ion permeability at smaller sheet size, other than interlayer spacing, and points to grain‐boundary density as a further structural parameter to consider in the transport behavior of laminar membranes.

## 3 Conclusion

In summary, we demonstrated that the lateral dimension of exfoliated layers plays a critical role in the crystallinity of laminar membranes. By systematically varying the sheet size, we quantified the change in polycrystalline structure of membranes, as evidenced by size‐dependent variations in mosaic distribution and domain size revealed through high‐resolution x‐ray diffraction analysis. These structural differences are reflected in the ion permeability, highlighting the importance of exfoliate size control in the design of laminar membranes, not only with phyllosilicates, but likely with all 2D materials. Our results emphasize the need to capture realistic pictures of membrane structure for self‐assembled laminar membranes. Polycrystalline structural analysis and exfoliate size control should be a mindful step to exercise while tailoring laminar membranes for ion separation applications.

## 4 Experimental Section/Methods

### 4.1 Materials and Chemicals

Raw vermiculite (2–3 mm), lithium chloride (≥99%), sodium chloride (≥99%), potassium chloride (≥99%), magnesium chloride (≥98%), yttrium(III) chloride hexahydrate (99.99% trace metals basis), potassium sulfate (≥99%), and potassium phosphate tribasic (≥97%) were purchased from Sigma–Aldrich. 1,6‐Hexanediamine (≥99%) was purchased from TCI America. PVDF membrane filter (hydrophilic, 0.22 µm pore size) was purchased from Millipore.

### 4.2 Preparation of Exfoliated Vermiculite Solutions and Membrane Fabrication

Bulk vermiculite was exfoliated using a modified ion‐exchange process reported previously [49]. Briefly, vermiculite pellets were added to a saturated NaCl solution and heated to 100°C for 24 h with vigorous stirring. The mixture was then filtered and rinsed with DI water to collect Na‐intercalated vermiculite (Na‐vermiculite). Na‐vermiculite was then added to 2 m LiCl solution heated again to 100°C for 24 h while stirring vigorously. After the reflux, the mixture was again filtered and rinsed with DI water to collect Li‐intercalated vermiculite (Li‐vermiculite). Li‐vermiculite was dispersed in DI water (ca. 4 g/L) and sonicated for 1, 4, 10, and 25 h to exfoliate into single‐layer nanosheet vermiculite solutions with different sizes (VS_0.6, VS_0.3, VS_0.2, and VS_0.1, respectively). Exfoliated vermiculite solutions were diluted in water (ca. 200 mg/L) and centrifuged at 2500 rpm for 10 min to remove unexfoliated vermiculite and large contaminants before use.

The 2D vermiculite laminar membranes were fabricated using vacuum filtration. In a typical process, cross‐linker solution (1 M of 1,6‐hexanediamine) was added to a vermiculite solution (50 µmol per mg of vermiculite) and sonicated for 30 min. The membrane mixture was filtered through a PVDF substrate and dried in air at 75°C. Dried vermiculite membranes on PVDF were peeled off from the substrate to be used as free‐standing vermiculite membranes with different sheet sizes.

### 4.3 Ion Permeation Measurement

The ion‐transport properties were characterized by diffusion dialysis in an H‐cell. The free‐standing cross‐linked VMs were pre‐soaked in DI water to remove impurities in the membrane. For each permeation experiment, 0.1 m of salt solutions (LiCl, MgCl_2_, YCl_3_, KCl, K_2_SO_4_, and K_3_PO_4_) were used as feed solutions. H‐cells were constantly stirred at 200 rpm to minimize concentration polarization. Permeate aliquots were collected over 24 h. Aliquots were diluted in 2% nitric acid solution and analyzed using ICP‐OES (iCAP PRO Duo, Thermo Scientific) to measure the concentration of cations.

The permeabilities for different ions were obtained under steady‐state diffusion conditions using Fick's first law for concentration‐driven diffusion process:

$$J_{i}=\frac{V}{A}\;\left(\frac{dc_{i}}{dt}\right)=-P_{i}\frac{dC_{i}}{dx}\;≅\;-P_{i}\frac{C_{i,0}}{\Delta x}$$

where *V* is the volume of the permeate, *A* is the effective membrane area, $\frac{dc_{i}}{dt}$ is the concentration gradient as a function of time. The permeability coefficient, *P_i_* was obtained by normalizing individual ion fluxes with Δ*x*, the membrane thickness measured using a micrometer, and *C*_*i*,0_, the driving force for mass transfer. Since the salt concentration in the permeate is much lower than that in the feed, the concentration difference across the membrane remains approximately equal to the feed concentration (Δ*C_i_* ≅*C*_*i*,0_).

### 4.4 Structural Characterization

The morphologies and height of the exfoliated vermiculite flakes were characterized by Atomic Force Microscopy (AFM) using a Cypher‐ES (Oxford Instruments) in AC‐air tapping mode and TITAN 300 AFM tips (Oxford Instruments). The results were analyzed with Igor Pro.

The morphologies of the surfaces and cross‐sections of vermiculite membranes were characterized by Scanning Electron Microscopy (SEM) using a JSM‐IT800HL (JEOL) with operating voltage of 3–5 kV. Vermiculite membranes were imaged directly without any surface treatment.

The x‐ray diffraction (XRD) patterns were obtained using a Rigaku benchtop x‐ray diffractometer with a Cu Kα radiation (λ = 1.5418 Å) operated at 40 kV and 44 mA.

Synchrotron‐based x‐ray diffraction rocking curve images were collected at beamline 7‐ID‐C at the Advanced Photon Source (APS), Argonne National Laboratory. The measurements were performed with a photon energy of 18 keV in a symmetric reflection geometry using an Eiger detector with a 75 µm pixel size at a distance of 700 mm from the sample, providing an angular sampling of 0.006° per pixel. The XRD data were obtained by mapping the intensities observed across the area detector to reciprocal space coordinates and then integrating the signals to produce an equivalent one‐dimensional diffraction intensity pattern. Because of the large number of pixels and high beam intensity, the measured signals had negligible statistical uncertainties. No changes in integrated intensity were observed over the course of the measurement.

### 4.5 X‐Ray Photoelectron Spectroscopy (XPS) Measurements

X‐ray photoelectron spectroscopy (XPS) measurements were performed using a Thermo Fisher Kα+ XPS with a micro‐focused monochromatic Al Kα (1487 eV) x‐ray source and 400 µm spot size. The survey scans used a pass energy of 200.0 eV with a step size of 1.000 eV. The high‐resolution scans used a pass energy of 50.0 eV with a step size of 0.100 eV. The depth profile measurements were performed by an Ar^+^ ion gun with an ion energy of 1000 eV and a sputtering rate of 0.29 nm/s that was calibrated to Ta_2_O_5_. Each of the 10 etch levels was etched for 10 s. The spectra were analyzed using the Thermo Fisher Avantage software (v6.11.2, Build 00003); all peaks were referenced to the adventitious carbon C 1s peak at 284.8 eV, and the high‐resolution peak deconvolution was performed using the Powell peak‐fitting algorithm with mixed Gaussian–Lorentzian line shapes and Smart background. Each presented spectrum is the average of five scans.

## Author Contributions

The manuscript was written through the contributions of all authors. All authors have given approval to the final version of the manuscript.

## Funding

This work was supported by the Advanced Materials for Energy‐Water Systems (AMEWS) Center, an Energy Frontier Research Center funded by the U.S. Department of Energy, Office of Science, Basic Energy Sciences. UChicago Argonne, LLC, Operator of Argonne National Laboratory (“Argonne”). Argonne, a U.S. Department of Energy Office of Science laboratory, is operated under Contract No. DE‐AC02‐06CH11357.

## Conflicts of Interest

The authors declare no conflicts of interest.

## Supporting information




**Supporting File**: advs76913‐sup‐0001‐SuppMat.pdf.

## Data Availability

The data that support the findings of this study are available from the corresponding author upon reasonable request.
