# Supplementary material for: Sheet‐Size‐Dependent Mosaicity of 2D Phyllosilicate Membranes
Source: Adv Sci (Weinh). 2026 Jul 31;13(60):e76913. doi: 10.1002/advs.76913 (PMC13427369; doi:10.1002/advs.76913)
Supplement: Supplementary file 1 [file ADVS-13-e76913-s001.pdf]

Supporting Information

Sheet-Size-Dependent Mosaicity of 2D Phyllosilicate Membranes

*Min A Kim, Paul A. Fenter, Sang Soo Lee, Katrina I. Sparks, Yining Liu, Jeffrey W. Elam, Seth B. Darling**

M. Kim, P. A. Fenter, K. I. Sparks, Y. Liu, J. W. Elam, S. B. Darling

Advanced Materials for Energy-Water Systems Energy Frontier Research Center, Argonne National Laboratory, Lemont, IL 60439, United States

M. Kim, P. A. Fenter, Sang Soo Lee, Y. Liu, S. B. Darling

Chemical Sciences and Engineering Division, Argonne National Laboratory, Lemont, IL 60439, United States

K. I. Sparks, J. W. Elam

Applied Materials Division, Argonne National Laboratory, Lemont, IL 60439, United States

K. I. Sparks, Y. Liu, S. B. Darling

Pritzker School of Molecular Engineering, University of Chicago, Chicago, IL 60637, United States

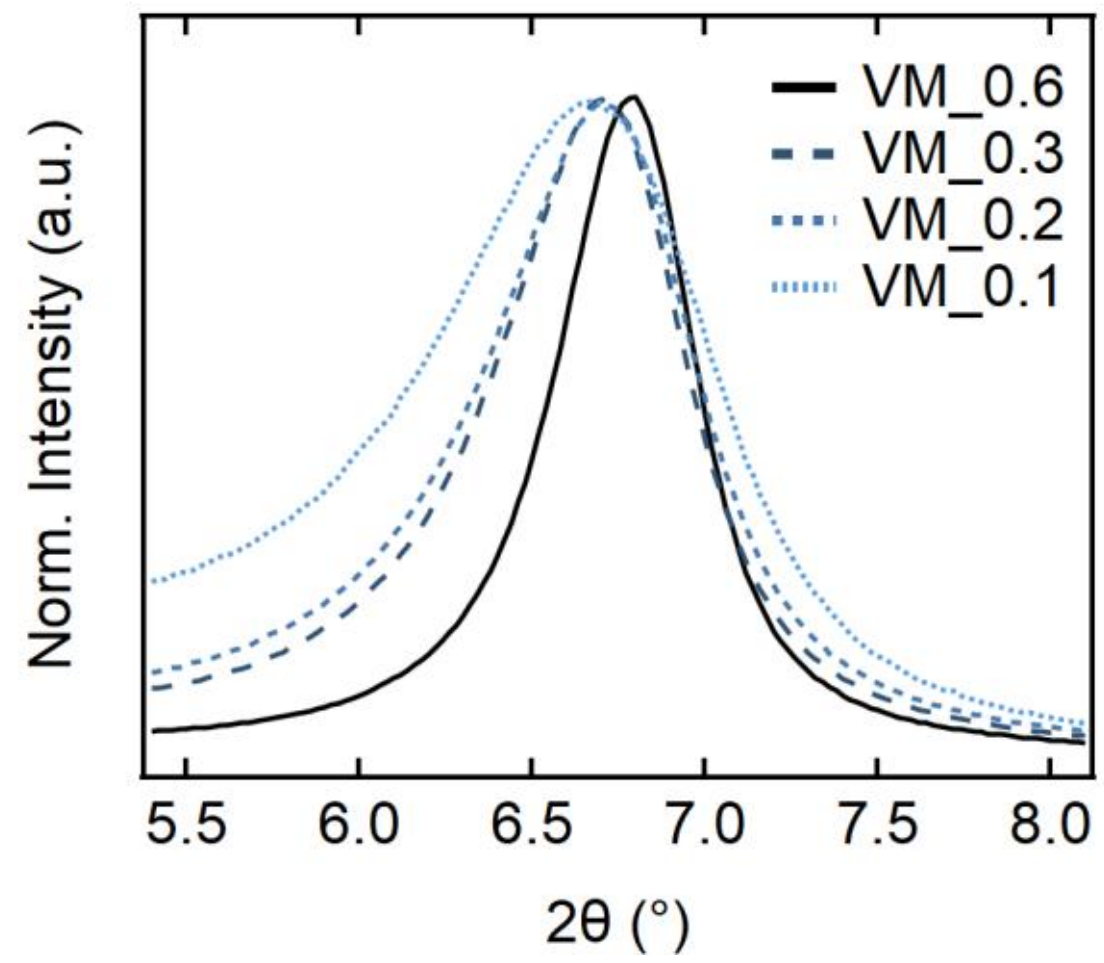

Figure S1. XRD 2θ scan of wet vermiculite membranes (VM_0.6, VM_0.3, VM_0.2, and VM_0.1) measured with a laboratory X-ray diffractometer. Membranes were soaked in water for 24 hours before the measurement.

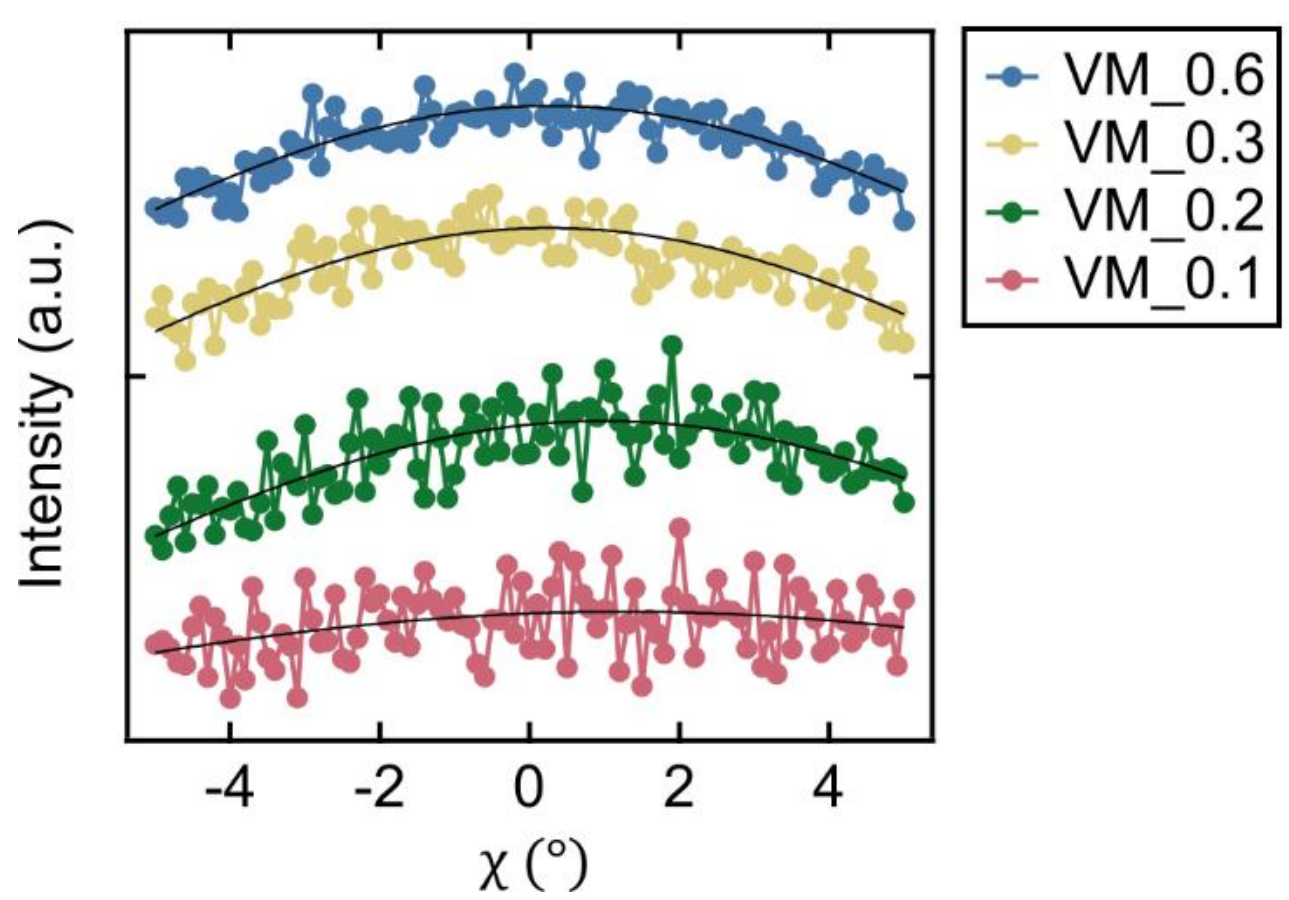

Figure S2. X-ray diffraction data for VMs with different sheet sizes (VM_0.6, VM_0.3, VM_0.2, and VM_0.1) obtained using a laboratory X-ray diffractometer. Mosaic distribution profile of the (001) reflection as a function of χ.

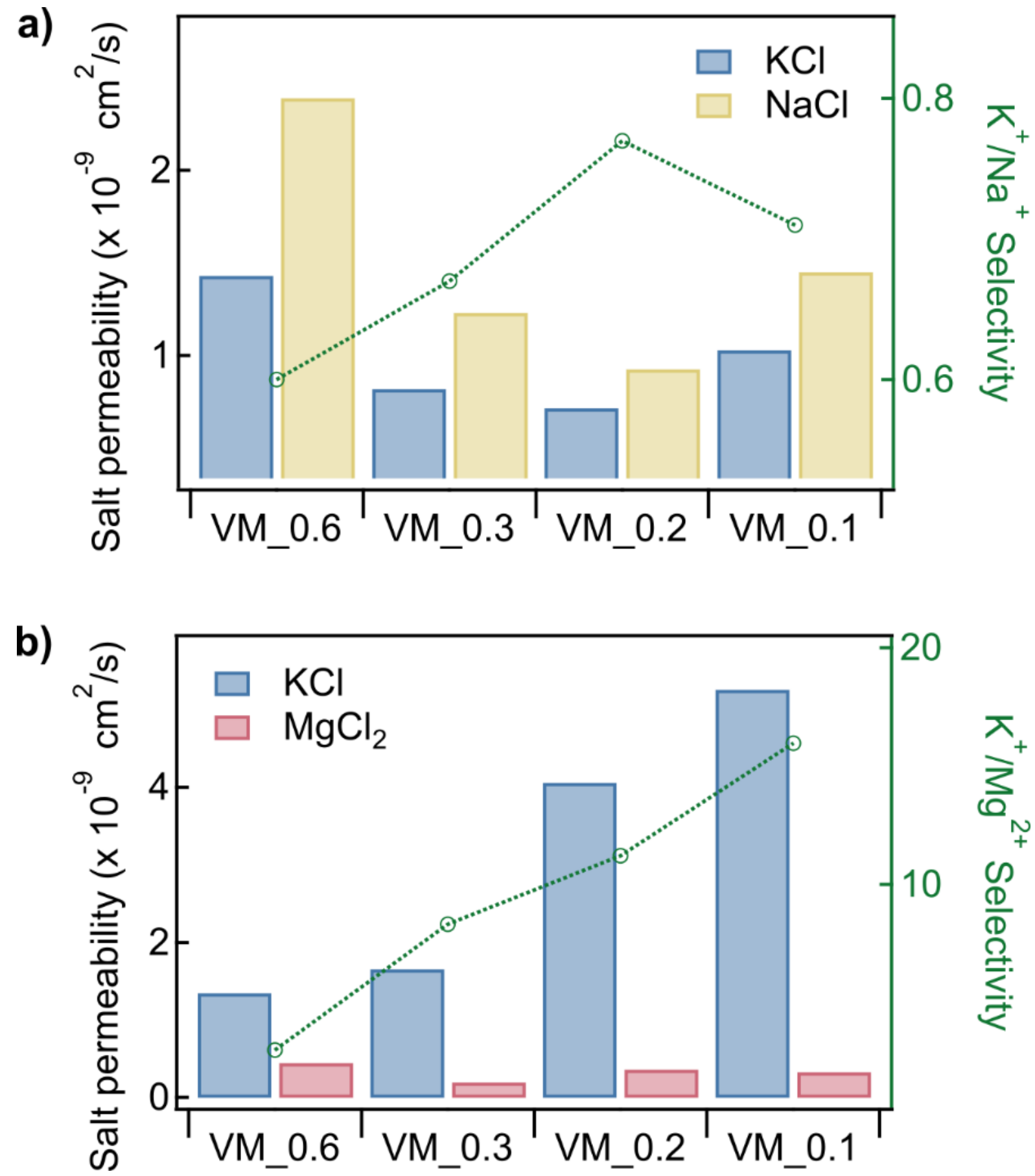

Figure S3. Binary solution ion permeability and selectivity for vermiculite membranes with different sheet sizes (VM_0.6, VM_0.3, VM_0.2, VM_0.1). a) K^+ and Na^+ permeability (bars, left axis) from a KCl/NaCl feed and the corresponding K^+/Na^+ selectivity (circle, right axis). b) K^+ and Mg^{2+} permeability (bars, left axis) from a $KCl/MgCl_2$ feed and the corresponding K^+/Mg^{2+} selectivity (circle, right axis).

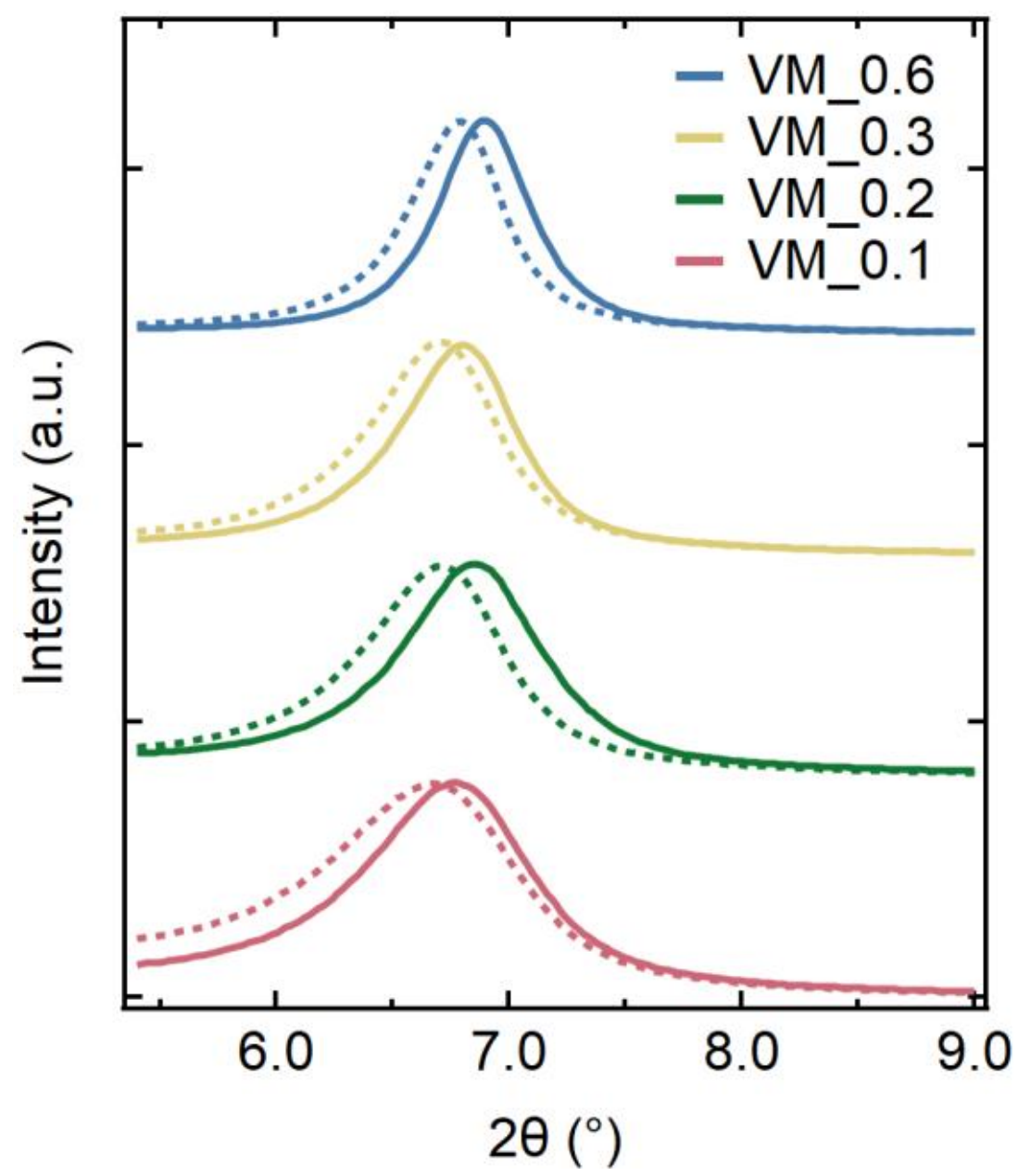

Figure S4. Dry and wet state of XRD scans for the vermiculite membranes (VM_0.6, VM_0.3, VM_0.2, VM_0.1), offset vertically for clarity. Solid lines are the dry membranes and dotted lines are the same membranes after 24 hours of water soaking.

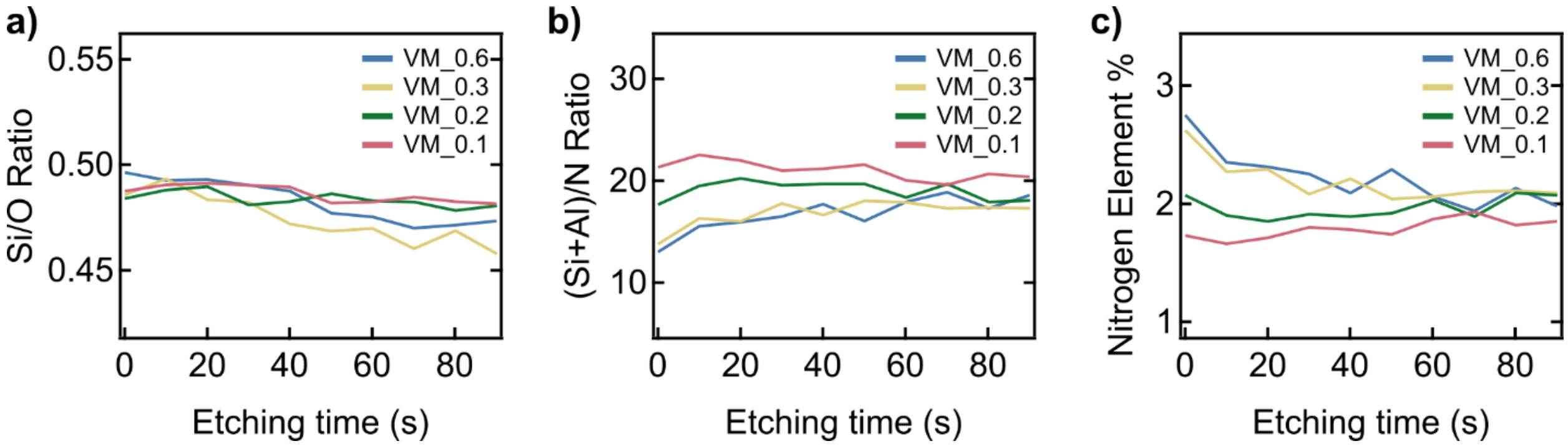

Figure S5. XPS depth profiles of the four membranes (VM_0.6, VM_0.3, VM_0.2, VM_0.1) as a function of Ar etching time. (a) Si/O ratio, (b) (Si+Al)/N atomic ratio, and (c) nitrogen percentage.

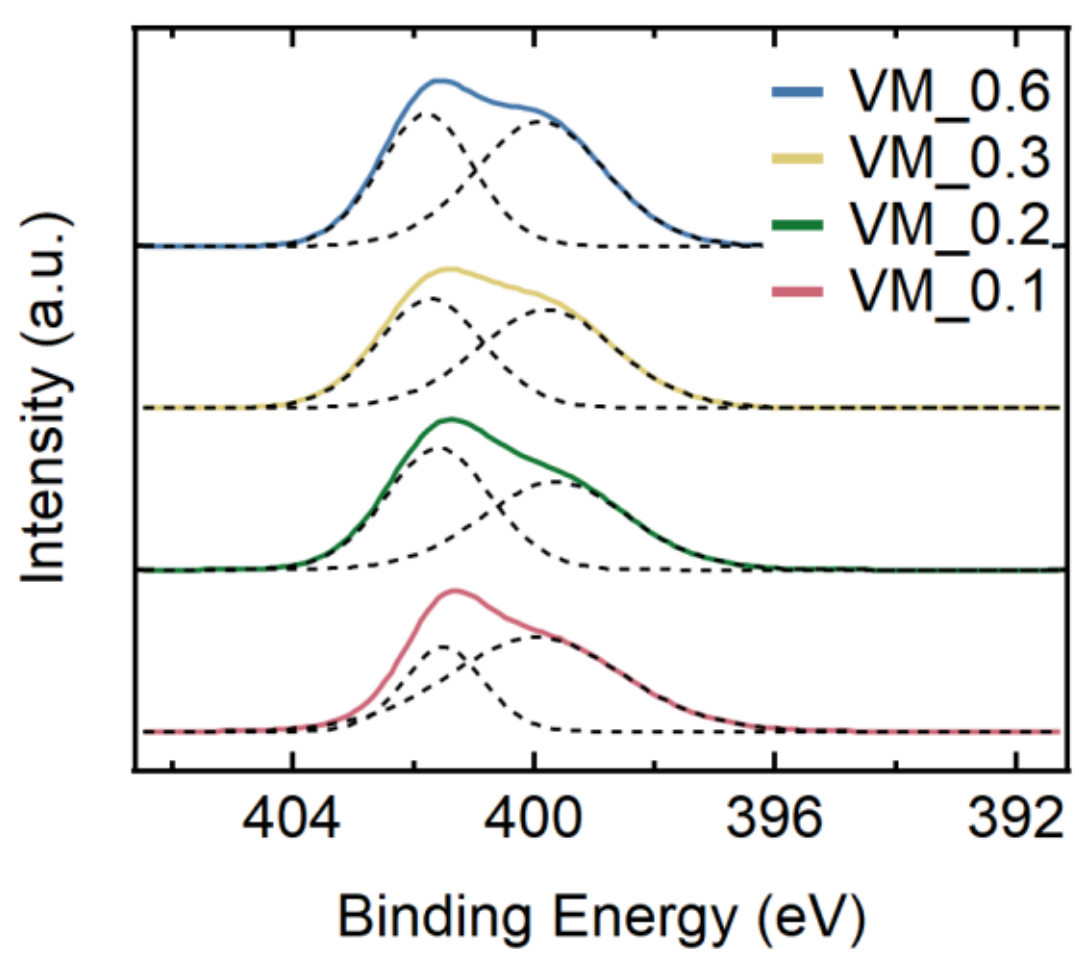

Figure S6. N 1s XPS spectra of the four membranes (VM_0.6, VM_0.3, VM_0.2, VM_0.1), offset vertically for clarity. Colored lines are the fitted envelopes, and dashed lines are the fitted components. Peaks near 399.8 and 401.6 eV are assigned to neutral and protonated amine of the hexanediamine cross-linker, respectively. The two components and their relative areas are comparable across the series, with peak positions varying by 0.3 eV.
